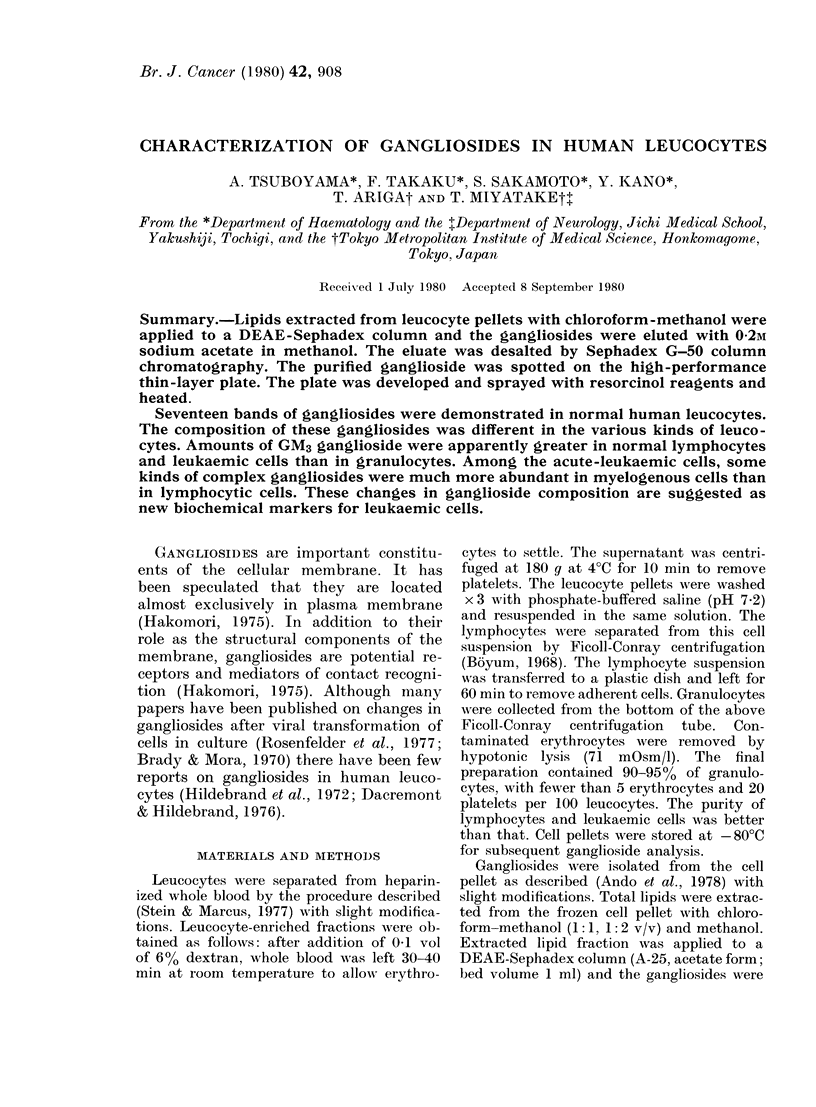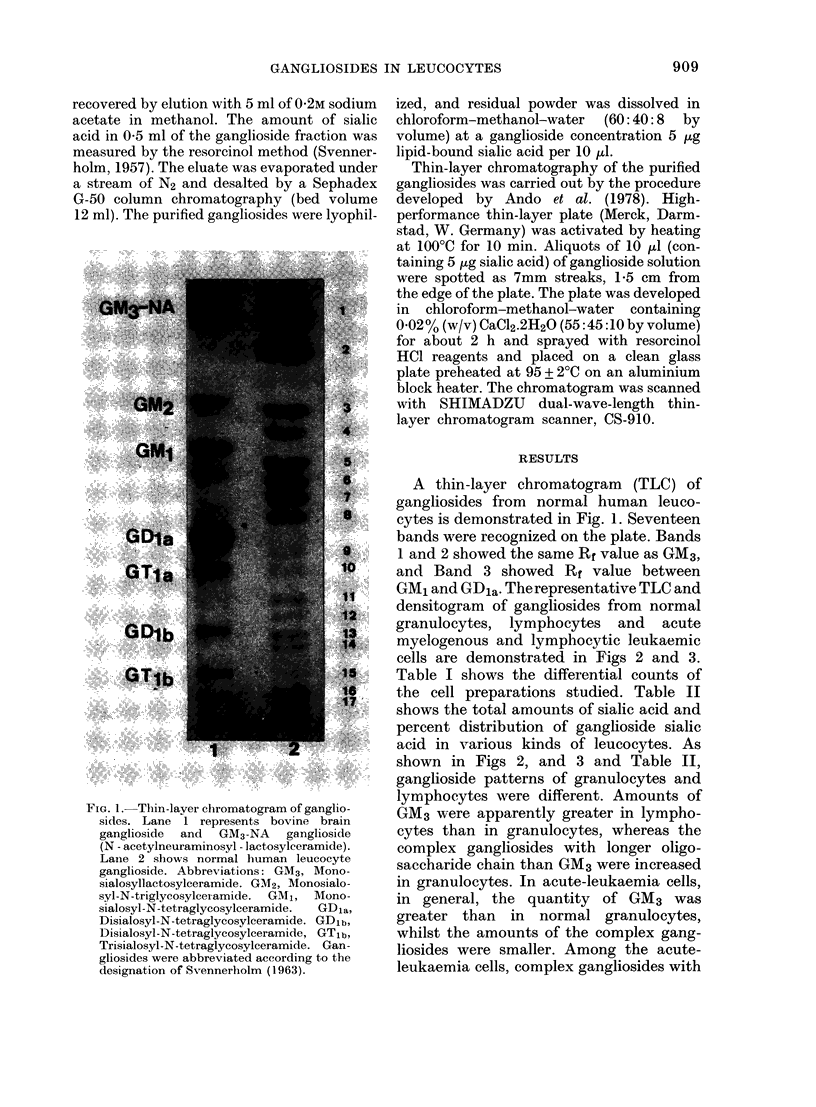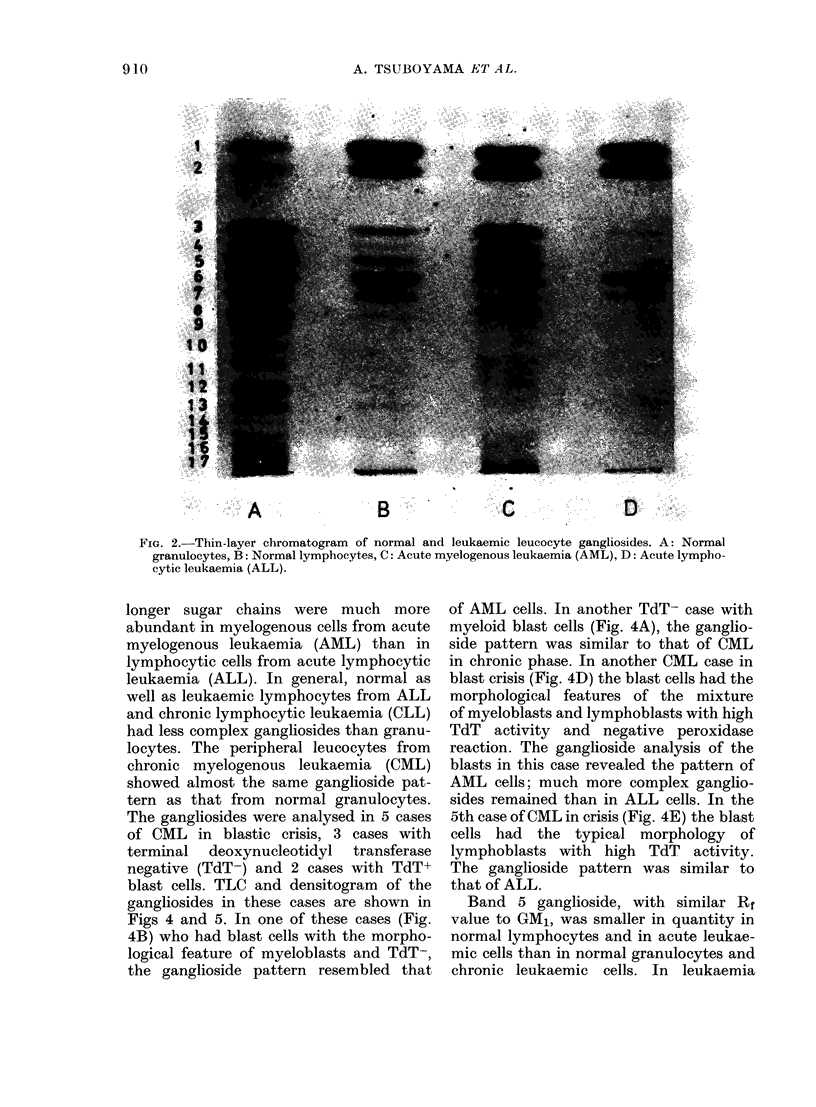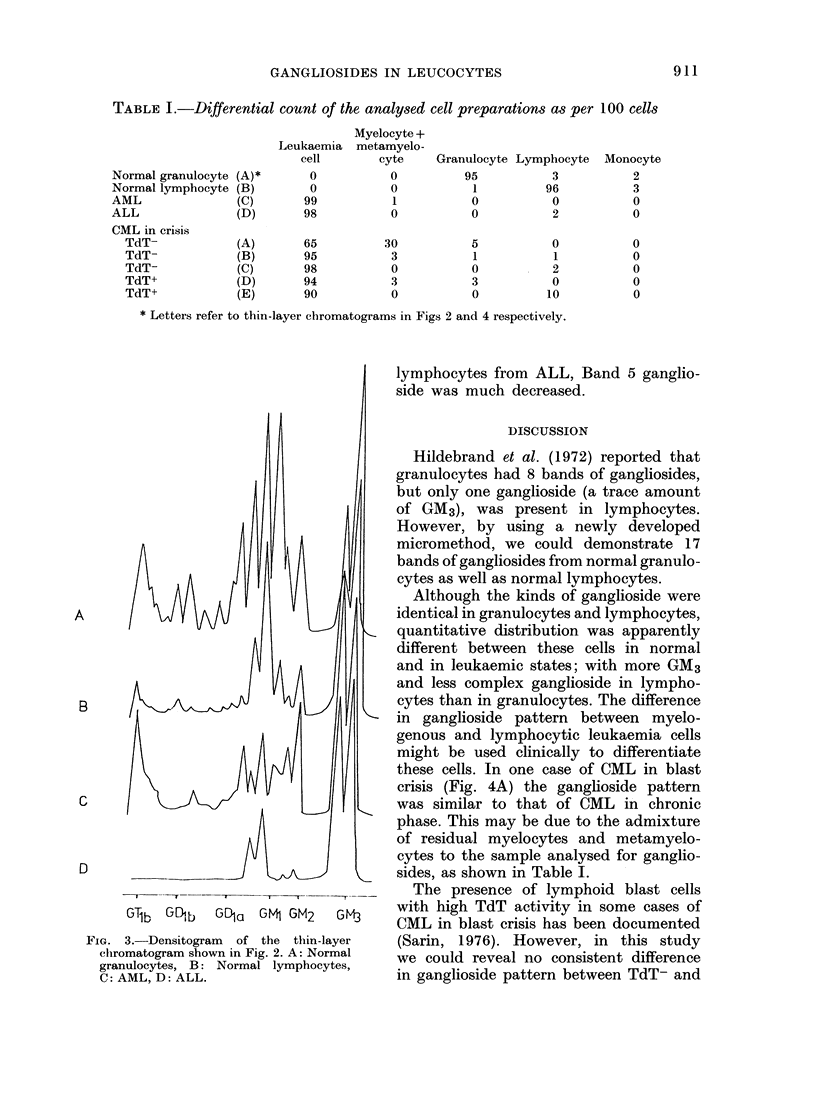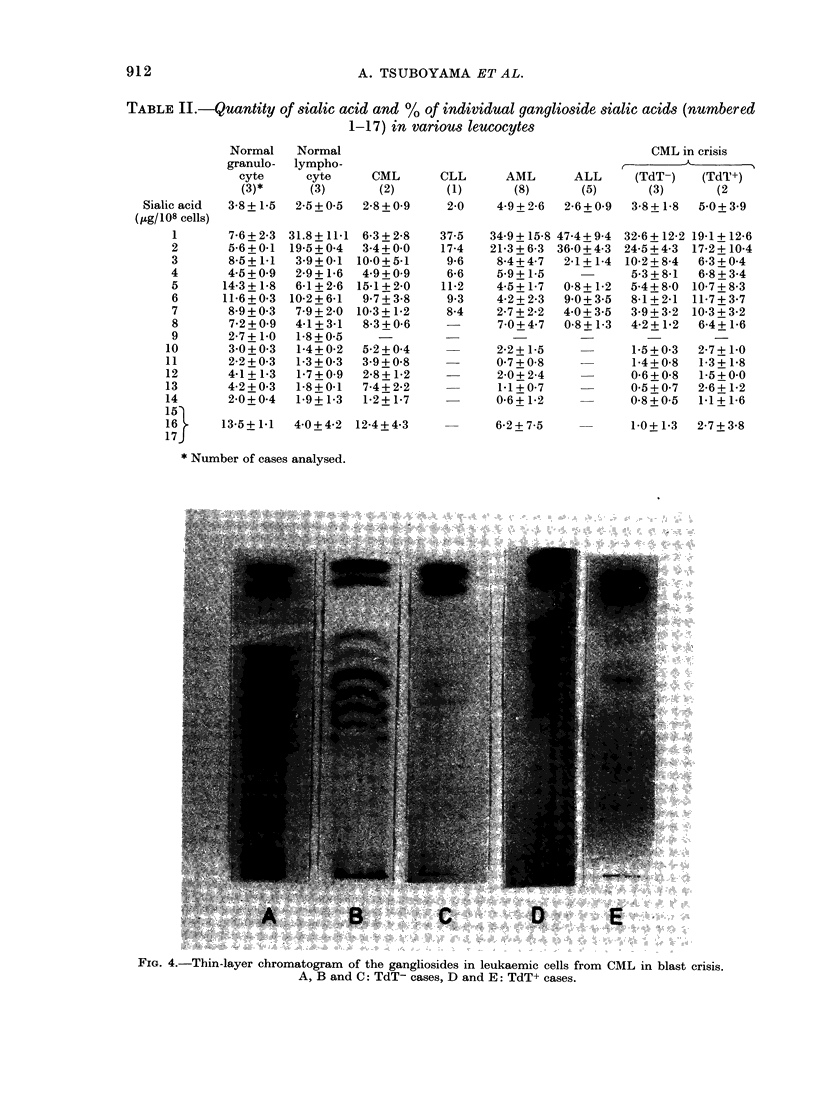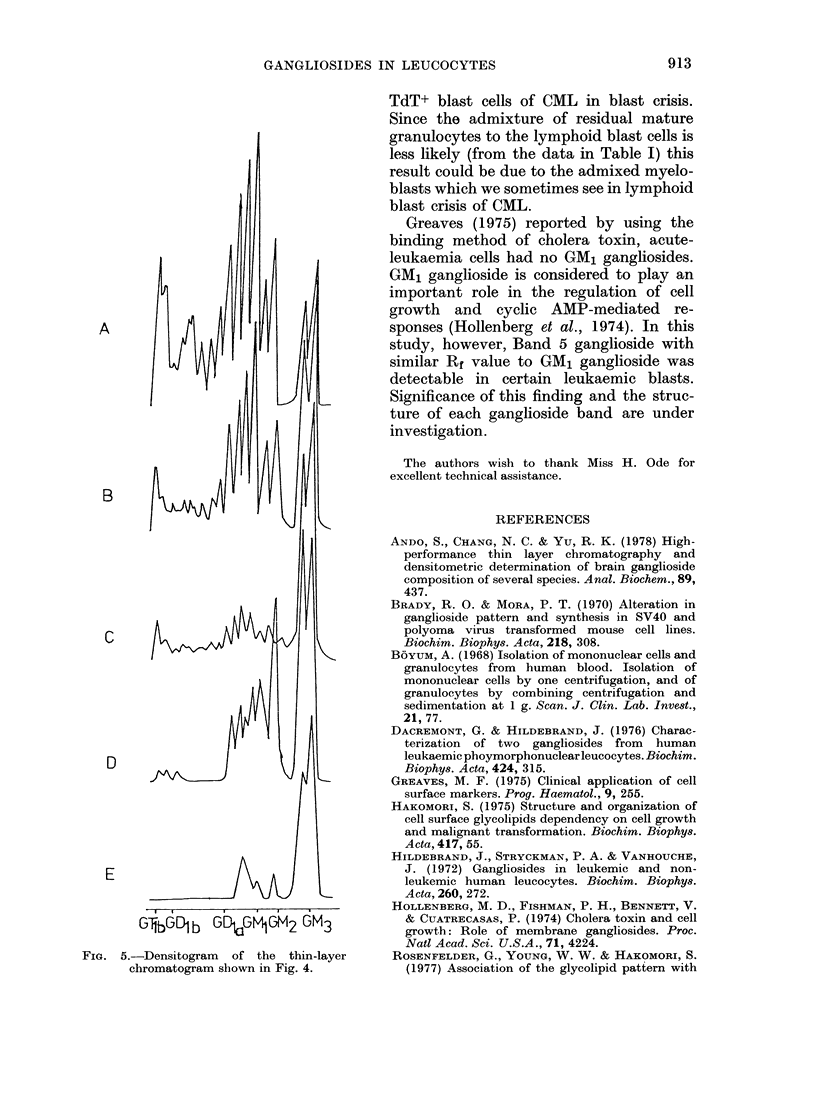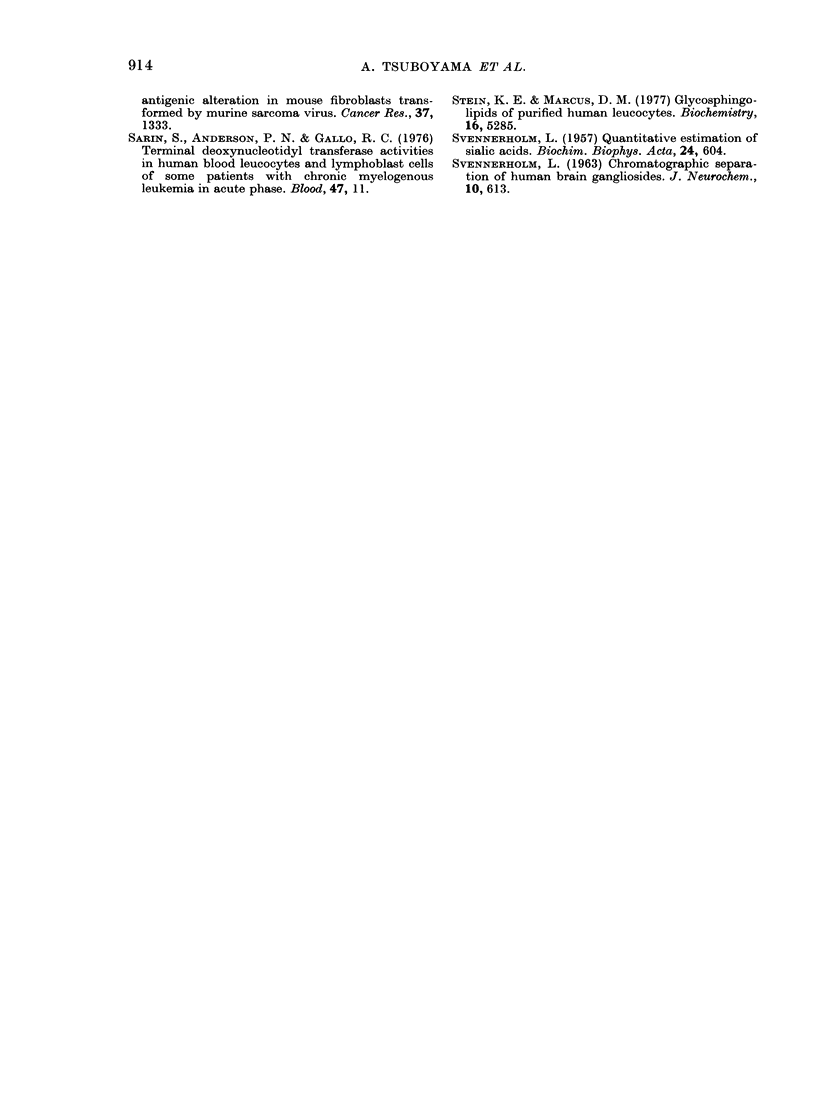# Characterization of gangliosides in human leucocytes.

**DOI:** 10.1038/bjc.1980.339

**Published:** 1980-12

**Authors:** A. Tsuboyama, F. Takaku, S. Sakamoto, Y. Kano, T. Ariga, T. Miyatake

## Abstract

**Images:**


*Br. J. Cancer* (1980) **42**, 908

### CHARACTERIZATION OF GANGLIOSIDES IN HUMAN LEUCOCYTES

A. TSUBOYAMA*, F. TAKAKU*, S. SAKAMOTO*, Y. KANO*, T. ARIGA† AND T. MIYATAKE†‡

*From the *Department of Haematology and the ‡Department of Neurology, Jichi Medical School, Yakushiji, Tochigi, and the †Tokyo Metropolitan Institute of Medical Science, Honkomagome, Tokyo, Japan*

Received 1 July 1980 Accepted 8 September 1980

**Summary.—Lipids extracted from leucocyte pellets with chloroform-methanol were applied to a DEAE-Sephadex column and the gangliosides were eluted with 0·2M sodium acetate in methanol. The eluate was desalted by Sephadex G–50 column chromatography. The purified ganglioside was spotted on the high-performance thin-layer plate. The plate was developed and sprayed with resorcinol reagents and heated.**

**Seventeen bands of gangliosides were demonstrated in normal human leucocytes. The composition of these gangliosides was different in the various kinds of leucocytes. Amounts of $GM_3$ ganglioside were apparently greater in normal lymphocytes and leukaemic cells than in granulocytes. Among the acute-leukaemic cells, some kinds of complex gangliosides were much more abundant in myelogenous cells than in lymphocytic cells. These changes in ganglioside composition are suggested as new biochemical markers for leukaemic cells.**

GANGLIOSIDES are important constituents of the cellular membrane. It has been speculated that they are located almost exclusively in plasma membrane (Hakomori, 1975). In addition to their role as the structural components of the membrane, gangliosides are potential receptors and mediators of contact recognition (Hakomori, 1975). Although many papers have been published on changes in gangliosides after viral transformation of cells in culture (Rosenfelder *et al.*, 1977; Brady & Mora, 1970) there have been few reports on gangliosides in human leucocytes (Hildebrand *et al.*, 1972; Dacremont & Hildebrand, 1976).

### MATERIALS AND METHODS

Leucocytes were separated from heparinized whole blood by the procedure described (Stein & Marcus, 1977) with slight modifications. Leucocyte-enriched fractions were obtained as follows: after addition of 0·1 vol of 6% dextran, whole blood was left 30–40 min at room temperature to allow erythrocytes to settle. The supernatant was centrifuged at 180 *g* at 4°C for 10 min to remove platelets. The leucocyte pellets were washed ×3 with phosphate-buffered saline (pH 7·2) and resuspended in the same solution. The lymphocytes were separated from this cell suspension by Ficoll-Conray centrifugation (Böyum, 1968). The lymphocyte suspension was transferred to a plastic dish and left for 60 min to remove adherent cells. Granulocytes were collected from the bottom of the above Ficoll-Conray centrifugation tube. Contaminated erythrocytes were removed by hypotonic lysis (71 mOsm/l). The final preparation contained 90–95% of granulocytes, with fewer than 5 erythrocytes and 20 platelets per 100 leucocytes. The purity of lymphocytes and leukaemic cells was better than that. Cell pellets were stored at −80°C for subsequent ganglioside analysis.

Gangliosides were isolated from the cell pellet as described (Ando *et al.*, 1978) with slight modifications. Total lipids were extracted from the frozen cell pellet with chloroform–methanol (1:1, 1:2 v/v) and methanol. Extracted lipid fraction was applied to a DEAE-Sephadex column (A-25, acetate form; bed volume 1 ml) and the gangliosides were recovered by elution with 5 ml of 0·2M sodium acetate in methanol. The amount of sialic acid in 0·5 ml of the ganglioside fraction was measured by the resorcinol method (Svennerholm, 1957). The eluate was evaporated under a stream of $N_2$ and desalted by a Sephadex G-50 column chromatography (bed volume 12 ml). The purified gangliosides were lyophilized, and residual powder was dissolved in chloroform–methanol–water (60:40:8 by volume) at a ganglioside concentration 5 $\mu$g lipid-bound sialic acid per 10 $\mu$l.

Thin-layer chromatography of the purified gangliosides was carried out by the procedure developed by Ando *et al.* (1978). High-performance thin-layer plate (Merck, Darmstad, W. Germany) was activated by heating at 100°C for 10 min. Aliquots of 10 $\mu$l (containing 5 $\mu$g sialic acid) of ganglioside solution were spotted as 7mm streaks, 1·5 cm from the edge of the plate. The plate was developed in chloroform–methanol–water containing 0·02% (w/v) $CaCl_2.2H_2O$ (55:45:10 by volume) for about 2 h and sprayed with resorcinol HCl reagents and placed on a clean glass plate preheated at $95 \pm 2$°C on an aluminium block heater. The chromatogram was scanned with SHIMADZU dual-wave-length thin-layer chromatogram scanner, CS-910.

FIG. 1.—Thin-layer chromatogram of gangliosides. Lane 1 represents bovine brain ganglioside and $GM_3$-NA ganglioside (N-acetylneuraminosyl-lactosylceramide). Lane 2 shows normal human leucocyte ganglioside. Abbreviations: $GM_3$, Monosialosyllactosylceramide. $GM_2$, Monosialosyl-N-triglycosylceramide. $GM_1$, Monosialosyl-N-tetraglycosylceramide. $GD_{1a}$, Disialosyl-N-tetraglycosylceramide. $GD_{1b}$, Disialosyl-N-tetraglycosylceramide, $GT_{1b}$, Trisialosyl-N-tetraglycosylceramide. Gangliosides were abbreviated according to the designation of Svennerholm (1963).

### RESULTS

A thin-layer chromatogram (TLC) of gangliosides from normal human leucocytes is demonstrated in Fig. 1. Seventeen bands were recognized on the plate. Bands 1 and 2 showed the same $R_f$ value as $GM_3$, and Band 3 showed $R_f$ value between $GM_1$ and $GD_{1a}$. The representative TLC and densitogram of gangliosides from normal granulocytes, lymphocytes and acute myelogenous and lymphocytic leukaemic cells are demonstrated in Figs 2 and 3. Table I shows the differential counts of the cell preparations studied. Table II shows the total amounts of sialic acid and percent distribution of ganglioside sialic acid in various kinds of leucocytes. As shown in Figs 2, and 3 and Table II, ganglioside patterns of granulocytes and lymphocytes were different. Amounts of $GM_3$ were apparently greater in lymphocytes than in granulocytes, whereas the complex gangliosides with longer oligosaccharide chain than $GM_3$ were increased in granulocytes. In acute-leukaemia cells, in general, the quantity of $GM_3$ was greater than in normal granulocytes, whilst the amounts of the complex gangliosides were smaller. Among the acute-leukaemia cells, complex gangliosides with

FIG. 2.—Thin-layer chromatogram of normal and leukaemic leucocyte gangliosides. A: Normal granulocytes, B: Normal lymphocytes, C: Acute myelogenous leukaemia (AML), D: Acute lymphocytic leukaemia (ALL).

longer sugar chains were much more abundant in myelogenous cells from acute myelogenous leukaemia (AML) than in lymphocytic cells from acute lymphocytic leukaemia (ALL). In general, normal as well as leukaemic lymphocytes from ALL and chronic lymphocytic leukaemia (CLL) had less complex gangliosides than granulocytes. The peripheral leucocytes from chronic myelogenous leukaemia (CML) showed almost the same ganglioside pattern as that from normal granulocytes. The gangliosides were analysed in 5 cases of CML in blastic crisis, 3 cases with terminal deoxynucleotidyl transferase negative ($TdT^-$) and 2 cases with $TdT^+$ blast cells. TLC and densitogram of the gangliosides in these cases are shown in Figs 4 and 5. In one of these cases (Fig. 4B) who had blast cells with the morphological feature of myeloblasts and $TdT^-$, the ganglioside pattern resembled that of AML cells. In another $TdT^-$ case with myeloid blast cells (Fig. 4A), the ganglioside pattern was similar to that of CML in chronic phase. In another CML case in blast crisis (Fig. 4D) the blast cells had the morphological features of the mixture of myeloblasts and lymphoblasts with high TdT activity and negative peroxidase reaction. The ganglioside analysis of the blasts in this case revealed the pattern of AML cells; much more complex gangliosides remained than in ALL cells. In the 5th case of CML in crisis (Fig. 4E) the blast cells had the typical morphology of lymphoblasts with high TdT activity. The ganglioside pattern was similar to that of ALL.

Band 5 ganglioside, with similar $R_f$ value to $GM_1$, was smaller in quantity in normal lymphocytes and in acute leukaemic cells than in normal granulocytes and chronic leukaemic cells. In leukaemia

TABLE I.—*Differential count of the analysed cell preparations as per* 100 *cells*

| | | Leukaemia cell | Myelocyte + metamyelo-cyte | Granulocyte | Lymphocyte | Monocyte |
|---|---|---|---|---|---|---|
| Normal granulocyte | (A)* | 0 | 0 | 95 | 3 | 2 |
| Normal lymphocyte | (B) | 0 | 0 | 1 | 96 | 3 |
| AML | (C) | 99 | 1 | 0 | 0 | 0 |
| ALL | (D) | 98 | 0 | 0 | 2 | 0 |
| CML in crisis | | | | | | |
| TdT$^-$ | (A) | 65 | 30 | 5 | 0 | 0 |
| TdT$^-$ | (B) | 95 | 3 | 1 | 1 | 0 |
| TdT$^-$ | (C) | 98 | 0 | 0 | 2 | 0 |
| TdT$^+$ | (D) | 94 | 3 | 3 | 0 | 0 |
| TdT$^+$ | (E) | 90 | 0 | 0 | 10 | 0 |

* Letters refer to thin-layer chromatograms in Figs 2 and 4 respectively.

FIG. 3.—Densitogram of the thin-layer chromatogram shown in Fig. 2. A: Normal granulocytes, B: Normal lymphocytes, C: AML, D: ALL.

lymphocytes from ALL, Band 5 ganglioside was much decreased.

### DISCUSSION

Hildebrand *et al.* (1972) reported that granulocytes had 8 bands of gangliosides, but only one ganglioside (a trace amount of $GM_3$), was present in lymphocytes. However, by using a newly developed micromethod, we could demonstrate 17 bands of gangliosides from normal granulocytes as well as normal lymphocytes.

Although the kinds of ganglioside were identical in granulocytes and lymphocytes, quantitative distribution was apparently different between these cells in normal and in leukaemic states; with more $GM_3$ and less complex ganglioside in lymphocytes than in granulocytes. The difference in ganglioside pattern between myelogenous and lymphocytic leukaemia cells might be used clinically to differentiate these cells. In one case of CML in blast crisis (Fig. 4A) the ganglioside pattern was similar to that of CML in chronic phase. This may be due to the admixture of residual myelocytes and metamyelocytes to the sample analysed for gangliosides, as shown in Table I.

The presence of lymphoid blast cells with high TdT activity in some cases of CML in blast crisis has been documented (Sarin, 1976). However, in this study we could reveal no consistent difference in ganglioside pattern between TdT$^-$ and

TABLE II.—*Quantity of sialic acid and % of individual ganglioside sialic acids (numbered 1–17) in various leucocytes*

| | Normal granulocyte (3)* | Normal lymphocyte (3) | CML (2) | CLL (1) | AML (8) | ALL (5) | CML in crisis (TdT−) (3) | CML in crisis (TdT+) (2 |
|---|---|---|---|---|---|---|---|---|
| Sialic acid (μg/10⁸ cells) | 3·8±1·5 | 2·5±0·5 | 2·8±0·9 | 2·0 | 4·9±2·6 | 2·6±0·9 | 3·8±1·8 | 5·0±3·9 |
| 1 | 7·6±2·3 | 31.8±11·1 | 6·3±2·8 | 37·5 | 34·9±15·8 | 47·4±9·4 | 32·6±12·2 | 19·1±12·6 |
| 2 | 5·6±0·1 | 19·5±0·4 | 3·4±0·0 | 17·4 | 21·3±6·3 | 36·0±4·3 | 24·5±4·3 | 17·2±10·4 |
| 3 | 8·5±1·1 | 3·9±0·1 | 10·0±5·1 | 9·6 | 8·4±4·7 | 2·1±1·4 | 10·2±8·4 | 6·3±0·4 |
| 4 | 4·5±0·9 | 2·9±1·6 | 4·9±0·9 | 6·6 | 5·9±1·5 | — | 5·3±8·1 | 6·8±3·4 |
| 5 | 14·3±1·8 | 6·1±2·6 | 15·1±2·0 | 11·2 | 4·5±1·7 | 0·8±1·2 | 5·4±8·0 | 10·7±8·3 |
| 6 | 11·6±0·3 | 10·2±6·1 | 9·7±3·8 | 9·3 | 4·2±2·3 | 9·0±3·5 | 8·1±2·1 | 11·7±3·7 |
| 7 | 8·9±0·3 | 7·9±2·0 | 10·3±1·2 | 8·4 | 2·7±2·2 | 4·0±3·5 | 3·9±3·2 | 10·3±3·2 |
| 8 | 7·2±0·9 | 4·1±3·1 | 8·3±0·6 | — | 7·0±4·7 | 0·8±1·3 | 4·2±1·2 | 6·4±1·6 |
| 9 | 2·7±1·0 | 1·8±0·5 | — | — | — | — | — | — |
| 10 | 3·0±0·3 | 1·4±0·2 | 5·2±0·4 | — | 2·2±1·5 | — | 1·5±0·3 | 2·7±1·0 |
| 11 | 2·2±0·3 | 1·3±0·3 | 3·9±0·8 | — | 0·7±0·8 | — | 1·4±0·8 | 1·3±1·8 |
| 12 | 4·1±1·3 | 1·7±0·9 | 2·8±1·2 | — | 2·0±2·4 | — | 0·6±0·8 | 1·5±0·0 |
| 13 | 4·2±0·3 | 1·8±0·1 | 7·4±2·2 | — | 1·1±0·7 | — | 0·5±0·7 | 2·6±1·2 |
| 14 | 2·0±0·4 | 1·9±1·3 | 1·2±1·7 | — | 0·6±1·2 | — | 0·8±0·5 | 1·1±1·6 |
| 15, 16, 17 | 13·5±1·1 | 4·0±4·2 | 12·4±4·3 | — | 6·2±7·5 | — | 1·0±1·3 | 2·7±3·8 |

* Number of cases analysed.

FIG. 4.—Thin-layer chromatogram of the gangliosides in leukaemic cells from CML in blast crisis. A, B and C: TdT− cases, D and E: TdT+ cases.

FIG. 5.—Densitogram of the thin-layer chromatogram shown in Fig. 4.

TdT+ blast cells of CML in blast crisis. Since the admixture of residual mature granulocytes to the lymphoid blast cells is less likely (from the data in Table I) this result could be due to the admixed myeloblasts which we sometimes see in lymphoid blast crisis of CML.

Greaves (1975) reported by using the binding method of cholera toxin, acute-leukaemia cells had no $GM_1$ gangliosides. $GM_1$ ganglioside is considered to play an important role in the regulation of cell growth and cyclic AMP-mediated responses (Hollenberg *et al.*, 1974). In this study, however, Band 5 ganglioside with similar $R_f$ value to $GM_1$ ganglioside was detectable in certain leukaemic blasts. Significance of this finding and the structure of each ganglioside band are under investigation.

The authors wish to thank Miss H. Ode for excellent technical assistance.

REFERENCES

ANDO, S., CHANG, N. C. & YU, R. K. (1978) High-performance thin layer chromatography and densitometric determination of brain ganglioside composition of several species. *Anal. Biochem.*, **89**, 437.

BRADY, R. O. & MORA, P. T. (1970) Alteration in ganglioside pattern and synthesis in SV40 and polyoma virus transformed mouse cell lines. *Biochim. Biophys. Acta*, **218**, 308.

BÖYUM, A. (1968) Isolation of mononuclear cells and granulocytes from human blood. Isolation of mononuclear cells by one centrifugation, and of granulocytes by combining centrifugation and sedimentation at 1 g. *Scan. J. Clin. Lab. Invest.*, **21**, 77.

DACREMONT, G. & HILDEBRAND, J. (1976) Characterization of two gangliosides from human leukaemic phoymorphonuclear leucocytes. *Biochim. Biophys. Acta*, **424**, 315.

GREAVES, M. F. (1975) Clinical application of cell surface markers. *Prog. Haematol.*, **9**, 255.

HAKOMORI, S. (1975) Structure and organization of cell surface glycolipids dependency on cell growth and malignant transformation. *Biochim. Biophys. Acta*, **417**, 55.

HILDEBRAND, J., STRYCKMAN, P. A. & VANHOUCHE, J. (1972) Gangliosides in leukemic and non-leukemic human leucocytes. *Biochim. Biophys. Acta*, **260**, 272.

HOLLENBERG, M. D., FISHMAN, P. H., BENNETT, V. & CUATRECASAS, P. (1974) Cholera toxin and cell growth: Role of membrane gangliosides. *Proc. Natl Acad. Sci. U.S.A.*, **71**, 4224.

ROSENFELDER, G., YOUNG, W. W. & HAKOMORI, S. (1977) Association of the glycolipid pattern with antigenic alteration in mouse fibroblasts transformed by murine sarcoma virus. *Cancer Res.*, **37**, 1333.

SARIN, S., ANDERSON, P. N. & GALLO, R. C. (1976) Terminal deoxynucleotidyl transferase activities in human blood leucocytes and lymphoblast cells of some patients with chronic myelogenous leukemia in acute phase. *Blood*, **47**, 11.

STEIN, K. E. & MARCUS, D. M. (1977) Glycosphingolipids of purified human leucocytes. *Biochemistry*, **16**, 5285.

SVENNERHOLM, L. (1957) Quantitative estimation of sialic acids. *Biochim. Biophys. Acta*, **24**, 604.

SVENNERHOLM, L. (1963) Chromatographic separation of human brain gangliosides. *J. Neurochem.*, **10**, 613.